# Establishing long-term survival and cure in young patients with Ewing's sarcoma

**DOI:** 10.1038/sj.bjc.6601955

**Published:** 2004-06-22

**Authors:** C L Weston, C Douglas, A W Craft, I J Lewis, D Machin

**Affiliations:** 1 UKCCSG, University of Leicester, Leicester, UK; 2 Department of Child Health, Royal Victoria Infirmary, Newcastle upon Tyne, UK; 3 St James' University Hospital, Leeds, UK; 4 Division of Clinical Trials and Epidemiological Sciences, National Cancer Centre, Singapore

**Keywords:** cure models, Ewing's sarcoma

## Abstract

This paper investigates the potential for long-term survivorship for young patients diagnosed with Ewing's sarcoma. Data are examined from two successive UKCCSG Ewing's Tumour studies (ET-1 and ET-2). Patients have been followed for up to 20 years. These studies had suggested that better 5-year survival with ET-2 over the earlier ET-1 was achieved by replacing cyclophosphamide by ifosfamide and increasing the dose of doxorubicin in a four-drug chemotherapy regimen. The updated hazard ratio, stratified for metastatic status at diagnosis, of 0.39 (95% confidence interval 0.12–0.61) confirmed the advantage of the ET-2 regimen in terms of overall survival. Cure models, based on the Weibull distribution, suggested that factors for long-term survival in addition to presence of metastases were age, primary site of tumour and study. Modelling identified the proportion cured with the ET-2 protocol as best at 70% in those who are under 10 years with a nonpelvic primary site and without metastatic disease. This contrasts to only 13% cure in those with the corresponding adverse prognostic indicators. Additionally, the risk of death remains greatest but relatively constant over the first 2 years postdiagnosis, and then declines to a lower but constant value for the next 3 years before reaching the ‘cure plateau’ at about 5 years. This investigation suggests that ‘cure’ is possible for patients with Ewing's sarcoma. This is established at approximately 5 years post diagnosis and the proportion cured depends on the presence of metastases, pelvic site and age at diagnosis.

The first UK national Ewing's Tumour Study (ET-1) commenced in 1978, following formation of the United Kingdom Children's Cancer Study Group (UKCCSG) in 1977. The objectives of the study were to establish a standard protocol for treatment of Ewing's tumour and to document tolerability, toxicity and complications. At that time, prognosis for patients with Ewing's tumour was poor with few patients surviving to 5 years (Nesbit, 1976). However, there was some suggestion that by adding chemotherapy regimens, utilising vincristine, actinomycin-D, cyclophosphamide and doxorubicin, with radiotherapy resulted in improved outcome (Rosen *et al*, 1974). It had also been suggested that older age, pelvic site, presence of metastatic disease and high lactic dehydrogenase (LDH) adversely affect outcome (Pomeroy and Johnson, 1975).

The nonrandomised and noncomparative ET-1 protocol was thus designed to utilise the maximum tolerated doses of these four drugs and radiotherapy in order to maximise their collective potential. The results of ET-1 were reported with a median follow-up of 11.2 years and the 5-year overall survival (OS) was 39% (95% confidence interval (CI) 31–47%) (Craft *et al*, 1997). The study concluded that chemotherapy and radiotherapy were indeed important in the management of Ewing's sarcoma, but acknowledged that better chemotherapy was needed if more patients were to survive.

At this time, new information was becoming available on the prognosis and treatment of patients with this disease (Nesbit *et al*, 1981; Jurgens *et al*, 1985). In a review of prognostic factors, the adverse influence of older age, pelvic site and presence of metastatic disease was confirmed (Glaubiger *et al*, 1980; Craft, 1985). In addition, male gender and increasing tumour volume were identified as possible indicators of poor outcome. Treatment strategies including doxorubicin (Nesbit *et al*, 1981), vincristine, doxorubicin, cyclophosphamide and actinomycin-D (Rosen *et al*, 1974) and ifosfamide (Demeocq *et al*, 1984; Bramwell *et al*, 1985; Gobel *et al*, 1986; Magrath *et al*, 1986) were suggested as regimens that could result in a higher response rate for patients with Ewing's tumour.

The aim of ET-2, albeit also nonrandomised, was to improve survival over ET-1 by retaining four drugs but replacing cyclophosphamide by ifosfamide and increasing the dose of doxorubicin. The results of ET-2 were reported, at a median follow-up of survivors of 4.8 years and gave a 5-year OS of 62% (95% CI 56–69%) (Craft *et al*, 1998). It was also noted that the 5-year survival of metastatic patients had increased from 9% in ET-1 to 23% in ET-2. As the characteristics of patients in ET-2 were similar to those of ET-1, it was concluded that the substantial improvement in survival was real. ET-2 also demonstrated that long-term survival is achievable in patients with Ewing's sarcoma and hence this raises the question of whether it is reasonable to claim ‘cure’ for those who survive beyond a particular time point from diagnosis.

The objective of this paper is to quantify the changing risk of patients following their diagnosis and to identify suitable time points where there are distinct changes in this risk with a view to identifying those patients who are truly long-term survivors. The proportion of long-term survivors is estimated using statistical cure models (CMs) (Sposto, 2002) and we also investigate whether patient characteristics are prognostic for long-term survival.

In addition, since we are using more complete follow-up data from both ET-1 and ET-2, an update of the earlier published reports is given.

## MATERIALS AND METHODS

### ET-1

ET-1 recruited patients between 1978 and 1986. Induction chemotherapy commenced with 2.0 mg m^−2^ (maximum 2.0 mg) vincristine, 50 mg m^−2^ (maximum 100 mg) doxorubicin and 1000 mg m^−2^ (maximum 1000 mg) cyclophosphamide with an additional 2.0 mg m^−2^ vincristine on days 8 and 15. At the discretion of the clinician, a second course was administered prior to local therapy for patients considered to have a good response to the first course of chemotherapy. Radiotherapy commenced at either day 15 or 36, depending on whether one or two courses of initial chemotherapy was given, and was dependent on site. Guidelines were 45 Gy for long bones to include the whole bone, 25 Gy for rib and 30 Gy for pelvic tumours. An additional boost of 10–15 Gy was recommended for all tumours. Doses for primary tumours of the spine depended on the length of the field, 40 Gy were recommended for fields of less than 15 cm and 32.5 Gy for longer fields, both given over 3 weeks. Surgery to the primary site was considered where clinically indicated, dependent on the site and age of the patient. During radiotherapy, 2.0 mg m^−2^ vincristine and 400 mg m^−2^ (maximum 600 mg) cyclophosphamide were given weekly. Following local therapy, chemotherapy with 2.0 mg m^−2^ vincristine, 50 mg m^−2^ doxorubicin and 600 mg m^−2^ cyclophosphamide was alternated every 3 weeks for 2 years with 2.0 mg m^−2^ vincristine, 1.4 mg m^−2^ (maximum 2.0 mg) actinomycin-D and 600 mg m^−2^ (maximum 1000 mg) cyclophosphamide. Patients aged less than 40 years of age with previously untreated biopsy-proven Ewing's tumour of the bone were eligible. Follow-up information is collected for 10 years from diagnosis. However, centres were specifically asked to provide an update on patient status in August 2002. More complete details of the study have been given previously (Craft *et al*, 1997).

### ET-2

ET-2 recruited patients between 1987 and 1993. This study utilised vincristine, 20 mg m^−2^ doxorubicin and ifosfamide, with actinomycin-D to be given when the total tolerable dose of doxorubicin had been administered. Chemotherapy was administered in three blocks. The first block consisted of four courses of 9 mg m^−2^ ifosfamide, 2 mg m^−2^ vincristine and 60 mg m^−2^ doxorubicin. Surgical excision of the primary tumour was then considered for all patients in an attempt to avoid or minimise use of radiotherapy. However, radiotherapy was recommended for patients with residual disease following surgery. For patients with macroscopic residual disease or for whom surgery was not possible, 55 Gy in 30 daily fractions over 6 weeks was recommended. For patients with microscopic residual disease, 45 Gy in 25 daily fractions over 5 weeks was suggested. For patients with lung metastases, whole-lung irradiation with 15 Gy in 10 daily fractions over 2 weeks was administered, with a local boost of 35 Gy in 23 fractions to sites of bulky disease. Patients also received 2.0 mg m^−2^ vincristine and 200 mg m^−2^ cyclophosphamide at weekly intervals during radiotherapy, although cyclophosphamide was omitted in patients with pelvic sites. Patients then received three weekly courses of 6 g m^−2^ ifosfamide, 2 mg m^−2^ vincristine, 60 mg m^−2^ doxorubicin followed by 10 weekly courses of 6 g m^−2^ ifosfamide, 2 mg m^−2^ vincristine, 1.5 mg m^−2^ actinomycin-D. Chemotherapy ceased at 52 weeks postdiagnosis. Patients aged less than 30 years with previously untreated biopsy-proven Ewing's tumour of the bone were eligible. Follow-up information is collected for 10 years from diagnosis. However, centres were specifically asked to provide an update on patient status in August 2002 and again in August 2003. More complete details of the study have been given previously (Craft *et al*, 1998).

### Statistical analysis

OS was defined as the time between date of diagnosis and date of death. Patients alive were censored at the date of last follow-up. OS was summarised using the Kaplan–Meier method and 95% CIs for OS at fixed time points were calculated using Greenwood's formula (Parmar and Machin, 1995). The difference in OS between ET-1 and ET-2, adjusted for the presence or absence of metastatic disease at diagnosis using the Cox proportional hazards model, was summarised by the hazard ratio (HR) and corresponding 95% CI. Prognostic factors included in the Cox models were based on those found to be significant in the original analyses (Craft *et al*, 1997; Craft *et al*, 1998). Calculations were made using the statistical procedures in SAS (2001).

### Cure models

In situations in which cures are not obtained, the proportion of patients who remain alive decays with time until all are dead. One statistical description for their survival is the Weibull model in which case the proportion alive, *W*(*t*), at time *t* is given by


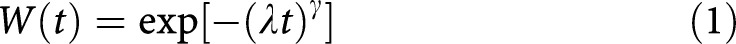


where *λ* and *γ* are constants that have to be estimated from the survival time data. These are termed the scale and shape parameters, respectively.

However, in circumstances where there may be a cure fraction of size *π*, the survival experience may be expressed as


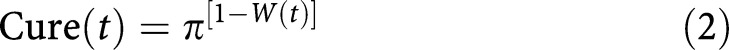


For this model when *t*=0, *W*(0)=exp[−(*λ* × 0)^*γ*^]=1 and so Cure(0)=*π*^[1−*W*(0)]^=*π*^0^=1 also. This indicates, as one would expect, that equations (1) and (2) imply that all are alive at time *t*=0. In contrast, when *t*=∞, *W*(∞)=exp[−(*λ* × ∞)^*γ*^]=0 and so all patients die under equation (1) while Cure(∞)=*π*^1^=*π* and so there are a proportion cured with equation (2).

**Figure 1:**
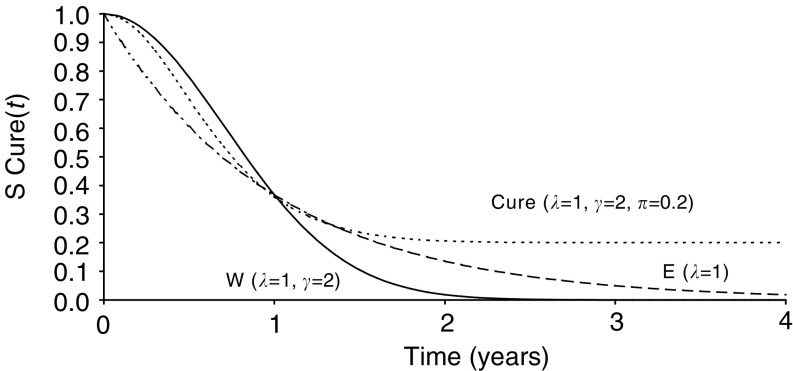
The proportion surviving with time according to the exponential, Weibull and cure models for *λ*=1, *γ*=2 and *π*=0.2.

If the Weibull model has shape parameter *γ*=1, then equation (1) becomes


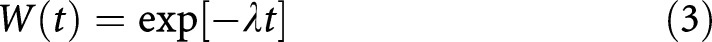


and this is the exponential model. In this model, *λ* corresponds to a constant hazard rate. This assumes that a single death rate applies to each patient irrespective of how long they have survived from the date of diagnosis. As examples, the survival experience described by models (1)–(3) is summarised in Figure 1

for *λ*=1, *γ*=2 and *π*=0.2.

The ‘cure’ fraction *π* of equation (2) may depend on the particular treatment the patient receives as well as upon patient-specific characteristics such as, for example, the presence or absence of metastases at diagnosis or their age. This can be expressed through a regression model for *π* such as


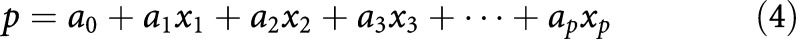


Here *x*_1_, *x*_2_,…, *x*_*p*_ are the (potentially prognostic) variables and *a*_0_, *a*_1_,…, *a*_*p*_ the corresponding regression coefficients to be estimated from the data.

Equation (2) is one form of several CMs described by Sposto (2002), which allows the possibility that *λ* and *γ* also depend on the potentially prognostic variables. A more detailed description of the CMs, together with a computer program that enables the parameters of such models, including *λ* and *γ* to be fitted, is available (Sposto, 2002).

### Changing hazard

Under the assumption of a Weibull distribution of survival times of equation (1), it follows that


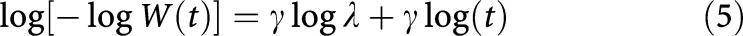


This is the equation of a straight line with intercept *γ* log *λ* and slope *γ*. Thus plots of log[−log(Kaplan–Meier estimate of survival at *t*)] against log(*t*) can be used to examine the changing hazard (risk of death) profile over time (Parmar and Machin, 1995). The objective was to see if the underlying hazard after a certain time was much lower than in the immediate post diagnosis period thereby (perhaps) indicating a ‘cure’ had been achieved for these patients. Any changes were determined by visual inspection of these plots.

## RESULTS

### Patient characteristics and overall survival

Between 1978 and 1986, 142 patients were entered onto the ET-1 study, 22 (15.5%) of whom had metastatic disease. The ET-2 study recruited 243 patients from 1987 to 1993, with 42 (17.3%) having metastatic disease. Patient characteristics were similar between the two studies (Table 1

**Table 1:** Characteristics of patients with Ewing's tumour of the bone recruited to ET-1 and ET-2

	**ET-1**			**ET-2**		
**Metastatic disease at diagnosis**	**No**	**Yes**	**Total**	**No**	**Yes**	**Total**
*Gender*						
Female	53 (37.3%)	15 (10.5%)	68 (47.9%)	87 (35.8%)	18 (7.4%)	105 (43.2%)
Male	67 (47.2%)	7 (5.0%)	74 (52.1%)	114 (46.9%)	24 (9.9%)	138 (56.8%)
						
*Age (years)*						
Mean	12.3	11.1	12.1	14.0	14.2	14.0
Range	1.8–34.0	4.6–15.4	1.8–34.0	1.9–27.7	1.5–27.0	1.5–27.7
						
*Site*						
Nonpelvic	97 (68.3%)	16 (11.3%)	113 (79.6%)	163 (67.1%)	31 (12.8%)	194 (79.8%)
Pelvic	23 (16.2%)	6 (4.2%)	29 (20.4%)	38 (15.6%)	11 (4.5%)	49 (20.2%)
						
*Metastases*						
None	120 (84.5%)			201 (82.7%)		
Lung		10 (7.0%)			22 (9.1%)	
Bone		7 (4.9%)			10 (4.1%)	
Lung and bone		1 (0.7%)			4 (1.6%)	
Liver		2 (0.14%			—	
Nodes		1^a^ (0.7%)			2^b^ (0.8%)	
Lung and other		1^c^ (0.7%)			1^d^ (0.4%)	
Bone and other		—			2^e^^f^ (0.8%)	
Lung, bone and other		—			1^g^ (0.4%)	
						
Total	120	22	142	201	42	243

a Para-aortic.

b Lymphatic.

c Mediastinum.

d Femoral nodes.

e Bone marrow, liver and lymph nodes.

f Bone marrow, liver and intracranial nodes.

g Bone marrow and pleura.

).

For ET-1 the median follow-up time is 17.0 years (range=0.3–24.0) and for ET-2 11.1 years (range=0.5–16.3). In all, 37% of alive patients have follow-up data of 2002 or later and a further 43% of alive patients have follow-up data of 1999 or later. It is to be expected that patients are seen in clinic less often as the time from diagnosis increases. The number of deaths in the two studies by metastatic status groups are given in Table 2

**Table 2:** CM and Cox regression estimates of survival for study and presence of metastases at diagnosis

**Model**	**Estimate**	**s.e.**	***z***	***P*-value**
*CM I*				
Intercept	0.090	0.110	0.82	0.415
Study				
ET-1	0			
ET-2	−0.779	0.141	−5.52	<0.001
Metastases				
No	0			
Yes	0.980	0.165	5.95	<0.001
Scale				
log *λ*	−1.432	0.066	−21.86	<0.001
*λ*	0.24			
Shape				
log *γ*	0.324	0.051	6.42	<0.001
*γ*	1.38			
				
*Cox I*				
Study				
ET-1	0			
ET-2	−0.712	0.142	5.00	<0.001
Metastases				
No	0			
Yes	0.954	0.164	5.80	<0.001

as are the 5-, 10- and 15-year survival rates. The corresponding OS curves are given in Figure 2

**Figure 2:**
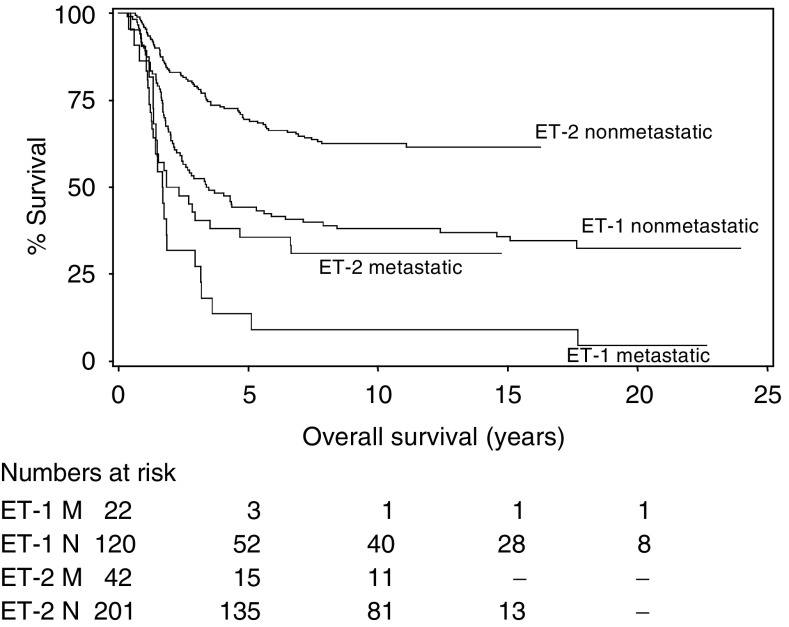
Kaplan–Meier estimates of the survival of patients in studies ET-1 and ET-2 by presence of metastases at diagnosis.

.

For both studies, there is clearly a worse outcome for those patients with metastases at diagnosis (Figure 2). The corresponding HR of ET-1 *vs* ET-2, stratified for metastases at diagnosis, is HR=0.39 (95% CI 0.12–0.61) (Table 2) and indicates a more favourable outcome for ET-2 patients.

### The fraction ‘cured’

Figure 2 suggests that the CM of equations (3) and (4) should include as variables the specific study (ET-1 or ET-2) and the presence or absence of metastases at diagnosis. Table 2 summarises the fit of the CM including these variables. The *P*-values for Study and Metastases are both <0.001, indicating a statistically significant contribution of these variables to the cure fraction. The corresponding predicted survival curves calculated from the CM of Table 3

**Table 3:** CM and Cox regression estimates of survival for study, presence of metastases at diagnosis, pelvic involvement and age

**Model**	**Estimate**	**s.e.**	***z***	***P*-value**
*CM II*				
Intercept	−0.224	0.168	−1.33	0.183
Study				
ET-1	0			
ET-2	−0.809	0.142	−5.71	<0.001
Metastases				
No	0			
Yes	0.878	0.167	5.27	<0.001
Pelvic				
No	0			
Yes	0.566	0.160	3.55	<0.001
Age ⩾10				
No	0			
Years				
Yes	0.314	0.171	1.84	0.065
Scale				
log *λ*	−1.448	0.066	−21.93	<0.001
*λ*	0.23			
Shape				
log *γ*	0.330	0.051	6.53	<0.001
*γ*	1.39			
				
*CM III*				
Intercept	−0.263	0.175	−1.51	0.132
Study				
ET-1	0			
ET-2	−0.904	0.154	−5.86	<0.001
Metastases				
No	0			
Yes	0.875	0.197	4.44	<0.001
Pelvic				
No	0			
Yes	0.614	0.178	3.45	0.001
Age ⩾10				
No	0			
Years				
Yes	0.426	0.180	2.37	0.018
Scale				
Intercept	−1.305	0.150	−8.72	<0.001
Study				
ET-1	0			
ET-2	0.267	0.130	2.05	0.041
Metastases				
No	0			
Yes	−0.069	0.244	−0.28	0.778
Pelvic				
No	0			
Yes	0.056	0.177	0.32	0.750
Age ⩾10				
No	0			
Years				
Yes	−0.338	0.148	−2.28	0.023
Shape				
Intercept	0.230	0.121	2.47	0.013
Study				
ET-1	0			
ET-2	0.135	0.110	1.23	0.218
Metastases				
No	0			
Yes	−0.116	0.175	−0.66	0.507
Pelvic				
No	0			
Yes	0.249	0.172	1.45	0.147
Age ⩾10				
No	0			
Years				
Yes	−0.041	0.128	−0.32	0.750
*Cox II*				
Study				
ET-1	0			
ET-2	−0.740	0.143	−5.18	<0.001
Metastases				
No	0			
Yes	0.861	0.166	5.17	<0.001
Pelvic				
No	0			
Yes	0.540	0.160	3.39	0.0007
Age ⩾10				
No	0			
Years				
Yes	0.297	0.170	1.74	0.0819

are shown in Figure 3

**Figure 3:**
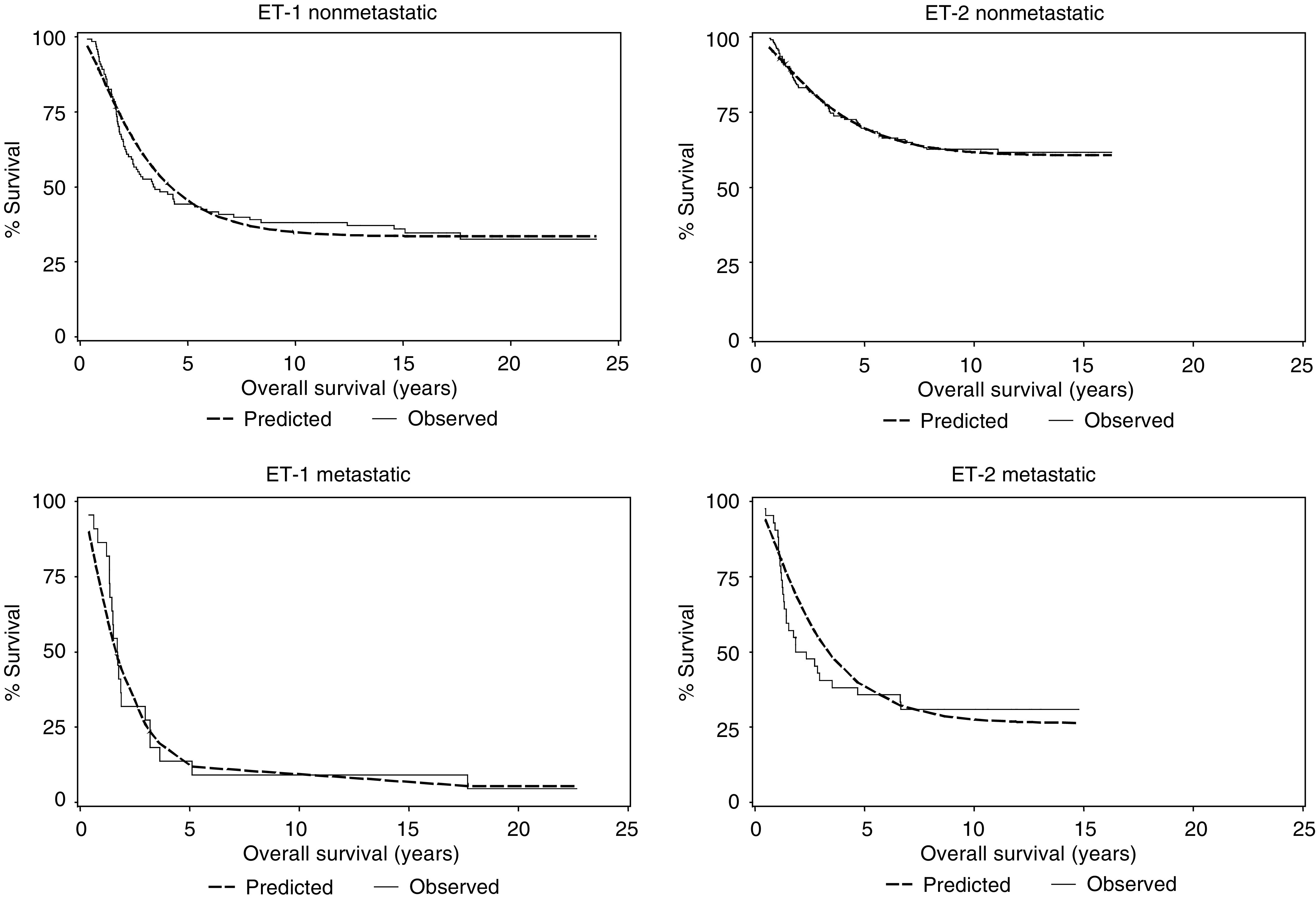
Kaplan–Meier estimates of the survival of patients in studies ET-1 and ET-2 by presence of metastases at diagnosis with the corresponding fitted CMs of Table 3.

superimposed on the Kaplan–Meier estimates.

The shape parameter *γ*=1.38 of the Weibull distribution is also statistically significant (*P*-value <0.001), confirming that this form of the survival times, rather than the exponential distribution, is indicated. Adding pelvic site and age to the CM gives the results corresponding to CM II in Table 3. There is little change in the estimates corresponding to Study (emphasised in bold within the table) or Metastases, and the values of the scale and shape parameters remain essentially unchanged. Nevertheless, the presence of pelvic disease is an adverse factor (*P*-value <0.001) in establishing cure and there is a suggestion that increasing age may also have an adverse role (*P*-value=0.065).

Adding the prognostic factors to the scale and shape of the CM gives the results corresponding to CM III in Table 3. This model suggests that age and study may be significant factors in the scale, but none of the factors appear to have a significant contribution to the shape parameter.

Table 4

**Table 4:** Estimated fraction cured (%) with 95% CIs by study, presence of metastasis at diagnosis, pelvic involvement and age

		**ET-1**		**ET-2**	
		**Metastatic**		**Metastatic**	
**Pelvis involved**	**Age (years)**	**No**	**Yes**	**No**	**Yes**
No	<10	45.0	14.6	70.1	42.5
		(32.9–6.3)	(2.5–36.9)	(52.0–82.4)	(11.3–71.5)
	⩾10	33.5	7.2	61.4	31.0
		(11.9–56.9)	(0.0–37.6)	(28.7–82.7)	(1.6–71.9)
					
Yes	<10	24.5	3.4	53.4	22.2
		(6.9–47.7)	(0.0–27.7)	(20.8–77.9)	(0.0–64.9)
	⩾10	14.6	1.0	42.4	12.7
		(0.0–48.4)	(0.0–28.4)	(4.9–78.3)	(0.0–65.4)

provides the corresponding estimates of the fraction cured calculated from CM II. This emphasises the difference in cure rates observed in the two studies, the influence of metastatic disease on lowering these rates and the worse outcomes for those with pelvic involvement and of greater age. Thus, the best cure fraction of 69.9% is observed in those of ET-2 with nonmetastatic, nonpelvic sites aged under 10 years, which reduces with this treatment to 12.6% in those with all the adverse prognostic factors and to only 1.0% in the corresponding patients in the ET-1 trial.

Table 5

**Table 5:** Estimated fraction cured (%) with 95% CIs by study, presence of metastasis at diagnosis, pelvic involvement and age, with these factors included in the scale and shape

		**ET-1**		**ET-2**	
		**Metastatic**		**Metastatic**	
**Pelvis involved**	**Age (years)**	**No**	**Yes**	**No**	**Yes**
No	<10	46.4	15.8	73.2	47.4
		(33.9–58.0)	(2.2–41.1)	(55.2–84.9)	(12.3–76.6)
	⩾10	30.8	6.0	62.1	31.9
		(9.5–55.6)	(0.0–38.4)	(27.5–83.9)	(1.1–75.1)
					
Yes	<10	24.2	3.3	56.2	25.1
		(5.9–49.1)	(0.0–31.3)	(21.1–80.8)	(0.4–70.6)
	⩾10	11.4	0.5	41.4	12.1
		(0.2–46.5)	(0.0–28.7)	(3.4–79.5)	(0.0–68.8)

provides the corresponding estimates of the fraction cured calculated from CM III. Again, this emphasises the difference in cure rates observed in the two studies, the influence of metastatic disease on lowering these rates and the worse outcomes for those with pelvic involvement and of greater age. However, adding prognostic factors to the scale and shape is seen to influence the estimates of the fraction cured. For example, the most extreme difference in the estimate of the fraction cured is 42.5% in CM II compared to 47.4% in CM III for ET-2 patients aged <10 years with a nonpelvic site and metastatic disease. However, the difference may be as small as 0.1% as seen for the ET-1 metastatic patients aged <10 years with a pelvic site.

For comparison purposes, we have included in Tables 2 and 3 the Cox proportional regression models utilising the same prognostic variables as we have used for the CMs (CMs I–III). It is clear from these that they too identify the earlier study, presence of metastases, pelvic involvement and older age as adverse characteristics with respect to survival time. However, these models cannot be used to estimate the fraction cured since they do not plateau, but behave in a similar way to the exponential and Weibull models of Figure 1.

### Establishing time to cure

**Figure 4:**
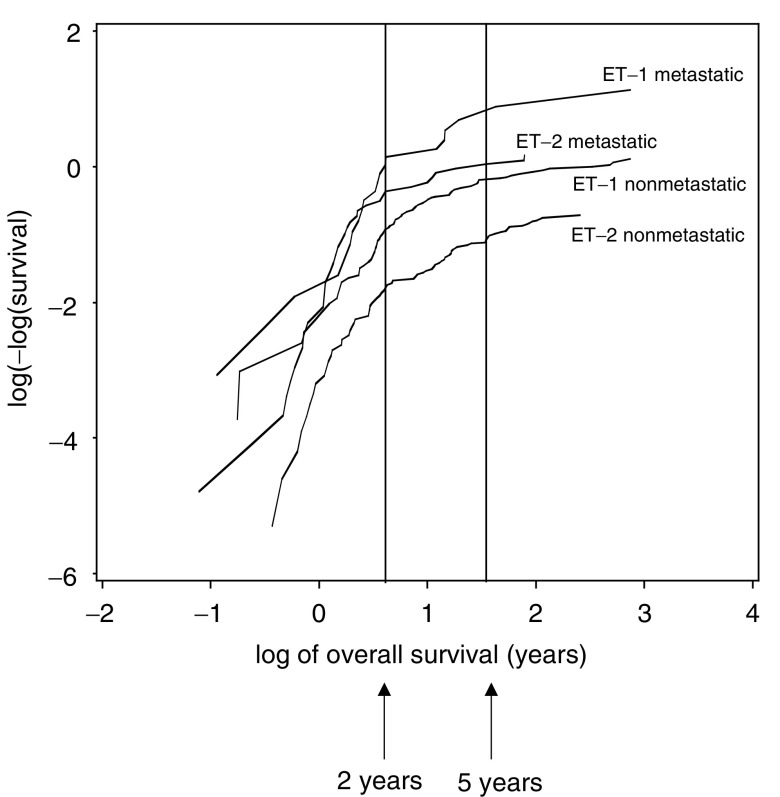
The log[−log(survival proportion)] against log(survival time) for studies ET-1 and ET-2 and by presence or absence of metastases at diagnosis.

The log[−log(survival)] plots for both ET-1 and ET-2 (Figure 4

) emphasise the improved survival with ET-2 and suggest that the hazards are approximately proportional in that the curves are essentially parallel. However, for both studies, there is an apparent decrease in the cumulative hazard rate at around 2 years and a further small decrease at around 5 years. This suggests a complex picture – an almost constant death rate postdiagnosis until approximately 2 years, a reduced but again near-constant death rate for the next 3 years and then a fall to the corresponding ‘plateau’. Although the CM does not require a formal definition of the time at which a long-term survivor can be considered as a cure, it is useful to have an indication of what may be considered as appropriate for this. On this basis, ‘cure’ is identified as survival beyond 5 years from diagnosis.

## DISCUSSION

The additional extra 5.8 years of follow-up for ET-1 and 6.3 years for ET-2 following publication of these studies (Craft *et al*, 1997, 1998) confirm the estimate of the overall 5-year survival of ET-1 as 39% and marginally increase that for ET-2 by 2%–64% (95% CI 58–70%). The HR, estimated using the Cox proportional hazards model adjusted for the presence of metastases at diagnosis, in favour of ET-2 is 0.39 (95% CI 0.12–0.61) and suggests a clear advantage to the newer regimen, although it is acknowledged that this is estimated from two successive (in time) studies and not from a randomised controlled trial of the two options. As such, this favourable outcome must be interpreted cautiously as there are many factors that could account for this improvement in survival. Although there is a strong possibility that the change in the chemotherapy between ET-1 and ET-2 did indeed contribute to the increase in survival, it must also be noted that the trials cover a large time interval (1978–1986 for ET-1, 1987–1993 for ET-2) and thus other factors may account for the apparent difference in survival between the two treatment regimens. For example, due to improved diagnostic techniques, patients may present with less advanced metastatic disease in ET-2 compared to ET-1. Changes in methods of local control may also have made a contribution. For example, in ET-2, patients with lung metastases were treated with whole-lung irradiation and this is likely to have contributed to the improved survival. Also, response to chemotherapy is now considered as an important prognostic factor, particularly in patients with localised disease. Early studies, such as ET-1 and ET-2, did not collect data relating to response, and thus it is difficult to assess whether improved survival is due to more patients showing a good response to the ET-2 chemotherapy regimen compared to ET-1. Therefore, although the enhancement in survival of ET-2 over ET-1 appears to be real, it is not possible to identify or quantify the precise reasons for improvement. This highlights the need to conduct randomised controlled trials, and this policy has been reflected in the third Ewing's sarcoma trial (EICESS 92) involving a wide European collaboration.

Despite the additional follow-up, there have been very few further ‘first’ relapses and/or deaths beyond those reported earlier. This suggests that ‘first’ events or deaths, from the Ewings' tumour itself, more than 5 years from diagnosis are rare. However, it should be recognised that neither study requested clinical information on any patient beyond 10 years from diagnosis; so it is possible that some relapses have gone unrecorded. Clearly, as time elapses, those patients remaining alive will approach the age-specific death rates of their contemporaries and so non-Ewings' sarcoma-related deaths may occur. In the young, road traffic accidents are a major cause. However, since the mortality of young patients is relatively small, their survival curve will appear as a ‘plateau’ for a number of years, followed by an eventual decline with increasing age. Once the decline commences, it will no longer be sensible to estimate a ‘cure’ fraction. Our analysis suggests that the risk of death is highest following diagnosis and remains constant for approximately 2 years, declines thereafter but again remains constant for 3 more years, and finally declines to a death rate consistent with cure.

In general, it is inadvisable to regard a plateau (such as those of Figure 2) as firm evidence that patients have been ‘cured’, since the level is unreliable unless a relatively large number of patients are still alive, and being followed up, for a survival duration considerably greater than the time at which this plateau begins (Peto *et al*, 1977). However, the CMs (Sposto, 2002) do permit a cure fraction to be estimated and also allow patient- and disease-specific factors prognostic for this fraction to be determined. Prognostic factors established for the cure fraction act independently of any effect they may have on early outcome.

Mixture models for cure have been proposed (Jones *et al*, 1981), which assume patients are either cured by treatment, that is, the disease is eradicated, or they are not, and only the latter would experience a recurrence after some time. However, Sposto (2002) argues that with combined modality treatment of long duration, this division into ‘eradicated’ and ‘not-eradicated’ at a relatively early stage postdiagnosis does not readily apply, since eradication of the disease, if it occurs, can occur at any time during the extended course of treatment. Although it is not necessarily always the case, the Cox models for the Ewing's tumour studies considered here identify the same factors as prognostic for survival time as do the CMs for ‘cure’. The CM is particularly likely to identify different prognostic factors to the Cox model where the nonproportional hazards are displayed between prognostic groups. Also, Cox models can only provide a satisfactory description of relative survival of the various groups in the earlier years following commencement of treatment, as they cannot plateau.

The Sposto (2002) CM includes the possibility that both the values of the scale and shape parameters depend on prognostic factors. Indeed, we noted in one subgroup (ET-2 metastatic patients less than 10 years with no pelvic involvement) that the estimate of the fraction cured was raised by as much as 4.9% when prognostic factors were included for the scale and shape parameters as well as for the cure fraction itself. Clearly, if firmly established, a potential difference in cure rates of this magnitude is clinically important and this emphasises the need to select the most appropriate model.

Although we have used and indeed estimated the proportion cured, the very term ‘cure’ in this context needs to be carefully examined as it is well recognised that these patients are at increased risk of developing a second malignancy and late toxic effects both of which may increase their risk of mortality. One possibility is to define cure in terms of event-free survival rather than merely survival alone, but to establish this it would require (perhaps unacceptable) detailed monitoring of patients for a very extended period.

In conclusion, we have established in these young patients with Ewings' sarcoma that the treatment regimen (in our case, ET-1 and ET-2), presence or absence of metastases, pelvic involvement and age are all indicative of the fraction eventually cured. We estimate that the cure fraction can range from as little as 1 to 70% dependent on regimen, patient- and disease-specific characteristics. There is also a suggestion (Table 4) that the ET-2 regimen has not only increased the cure fractions but has also reduced the proportionate gap in the cure fraction between metastatic and nonmetastatic patients from approximately 1/4 to 1/2 in the four pelvis involved by age groups. As survival rates continue to improve, long-term survival and cure are becoming increasingly important end points when planning clinical trials in patients with Ewing's sarcoma.
